# Books

**Published:** 1882-06

**Authors:** 


### Books.

---

A System of Surgery, Theoretical and Practical. In Treatises by Various Authors. Edited by T. Holmes, M.A., Cantab First American from Second English Edition. Revised by Jno. H. Packard, A.M., M.D., assisted by a large corps of American Surgeons. Henry C. Lea's Son & Co.: Philadelphia. 1882.

This is the third volume of Holmes' work. We have already noted the first and second most favorably. This one is not inferior to either of the preceding. The English contributors are among the most noted surgeons. In the list of American revisers we find the names of J. Solis Cohen, Thomas M. Markoe, E. H. Bradford, P. S. Conner, Roberts Bartholow, Hunter McGuire, J. C. Reeve, Chas. T. Hunter, John H. Packard, T. G. Morton, Theodore A. McGraw, Arthur Van Harlingen, Joseph Leidy, Samuel Ashhurst and Norton Folsom.

The first part, of 115 pages, by several different authors. has all been revised by J. Solis Cohen, M.D. It is devoted to the various surgical affections of the respiratory organs. His work is well done. We notice in the treatise on bronchocele that Dr. Graves suggests that the *globus hystericus* may not really always be a nervous affection, but may depend on a swelling (temporary) of the thyroid gland.

The chapter on apnœa (asphyxia), in its various forms, or depending on its various causes, is well given. The various methods of artificial respiration are described and illustrated. Preference seems to be given, very properly we think, to that method known as Silvester's. In the child the mouth to mouth method is recommended. We cannot agree, however, from our own experience, that Silvester's method is ineffectual in this class of patients. A hand bellows is also recommended. Directions are given for introducing, in some cases, a catheter or tube through the mouth into the trachea. We suggest it would be easier to open the larynx. It is recommended that in the Silvester method the movement be repeated as frequently as even forty times per minute. We must say this is too frequent, if not impossible, for proper execution.

Diseases of the bones are well treated by T. Holmes, Esq., and revised by Thos. M. Markoe, M.D. The chapters on the joints, the spine, and on orthopædic surgery, are revised by E. H. Bradford, M D.; the last is only moderately well done. The chapter on the nervous system, revised by Roberts Bartholow, is not so elaborate as it might well be. Dr. Hunter McGuire does a good work in his revision of gunshot wounds. He treats these injuries of the abdomen fully and well. In the chapter on operative and minor surgery, we find that "ether is still extensively used as an anæsthetic in America, but in Europe chloroform is generally preferred." In all accidents from anæsthetics "the three great remedies are traction of the tongue, inversion of the body and artificial respiration." A number of anæsthetics are treated of. The chapters on amputations, ligations and plastic surgery are carefully presented. Under minor surgery, bandaging is rather carelessly taught. The diseases of the breast are assigned to a separate chapter—nothing is offered beyond what the profession is already familiar with. In cases where malignancy is suspected prompt operation by excision is urged. More than one hundred pages are devoted to skin diseases. The author insists that chrysarobin is the proper name for what is generally known as chrysophanic acid. He regards it as the best simple remedy for psoriasis. The chapters on surgical diseases of children are of much interest. Surgical diagnosis and regional surgery are treated together in an entertaining manner. Many other divisions are given, which we have not space to note. The book closes with fifty pages on hospitals, their site, construction, management, nursing, diet, etc. At the close is a good index to the volume, and also a fairly good one for the whole work.

On the whole, this volume is equal to the first and second. We again commend the work as one of great value, well covering the vast field of surgery.

---

Lectures on Electricity (Dynamic and Franklinic), in its Relations to Medicine and Surgery. By A. D. Rockwell, A.M., M.D. New York: William Wood & Co. 1881.

This manual, of one hundred and twenty pages, consists of eight lectures delivered by the author, and first published in consecutive numbers of the *Virginia Medical Monthly.* This is the second edition of the work, and the additions of importance relate to descriptions of the "galvanic accumulator," a device for storing electricity for surgical uses; the "induction balance," an ingenious piece of mechanism intended to indicate the position of bullets in the body, and a full account of Franklinic electricity.

The book is similar to other elementary treatises on this subject already before the public, but the author tells us that this brochure is offered in consideration of the fact that, in addition to its general survey of electro-therapeutics, it gives some points that are not discussed in the present edition of his larger work; and again, that while a number of books on this subject, equally concise, have been issued, in none of them have his methods of general faridization and central galvanization been given any adequate consideration, and by some not even mentioned.

There is, perhaps, a decided difference of opinion among electro-therapeutists concerning the actual value of these methods; hence, possibly, the inattention bestowed upon them by some writers.

The book is well printed in large, clear type, neatly bound and amply illustrated. To the student of medicine and to the general practitioner it will prove a useful aid in the study of the subject and a valuable guide in the application of electricity in its several forms, to the treatment of disease.

---

Annual Report of the Department of Agriculture [United States], for the year 1880. Washington: Government Printing Office. 1881. Through the courtesy of U. S. Senator, Hon. Joseph E. Brown.

This volume of 672 pages includes among its contents the reports of the commissioners, of the chemist, of the statistician, of the entomologist, of the botanist on grasses, of Dr. Salmon on swine-plague and chicken-cholera, of Dr. Law on swine-plague, of Dr. Detmers on swine-plague, of Dr. Lyman on contagious pleuro-pneumonia; also reports on aphthous fever, or foot and mouth disease, investigation into Texas cattle fever and on bronchitis in cattle, by Prof. W. Williams.

The book is profusely and well illustrated, and, besides much practical information, it contains also the result of many painstaking, scientific investigations into some of the important subjects discussed in the several reports.

---

AN INDEX OF SURGERY. Being a concise classification of the main facts and theories of surgery for the use of senior students and others. By C. B. Keetley, F.R.C.S. New York: Bermingham & Co. 1882. The same from William Wood & Co., New York. 1882.

This book was designed for the use of senior students—to facilitate a review of the subject of which it treats before and in anticipation of final examination. The work of the author has been carefully and faithfully done, and the result is a book useful not alone to students, but valuable also to the practitioner; affording him a convenient compilation of practical surgical knowledge in a most convenient shape for ready reference. The contents are arranged alphabetically, and it is very properly styled "an index of surgery."

The two editions are very similar. The Bermingham edition contains 208 pages, is more closely printed, in finer type and on rather better paper. The Wood edition contains an index of names, wanting in the other, is printed in very plain, clear type, covering 320 pages, but on inferior paper. We do not hesitate to commend the book for just what it claims to be.

